# Exploring cell-type associations of rare disease phenotypes using non-diseased cell atlases

**DOI:** 10.1093/bib/bbag426

**Published:** 2026-08-06

**Authors:** Jorge Novoa, Florencio Pazos, Monica Chagoyen

**Affiliations:** Computational Systems Biology Group. National Center for Biotechnology (CNB-CSIC), Darwin 3, 28049 Madrid, Spain; Computational Systems Biology Group. National Center for Biotechnology (CNB-CSIC), Darwin 3, 28049 Madrid, Spain; Systems Biomedicine Lab. Cajal Neuroscience Center (CNC-CSIC), Avda. de Leon 1, 28805 Alcala de Henares, Spain

**Keywords:** rare diseases, scRNA-seq, human cell atlases

## Abstract

Rare diseases individually affect few patients but collectively impose a substantial global health burden. Many have a genetic origin, yet the cellular contexts in which disease genes exert their effects often remain unclear. Direct molecular investigation of disease-relevant tissues is often infeasible owing to small patient populations and frequent congenital or pediatric onset, limiting access to patient-derived samples. Here we investigate whether existing healthy human single-cell atlases can help identify candidate cellular contexts associated with rare disease phenotypes. Specifically, we test the hypothesis that cells expressing more genes linked to a phenotype than expected from their overall transcriptional activity may represent contexts particularly susceptible to disruption. Applied to more than 1300 phenotypes across multiple tissues, the analysis shows partial concordance with literature-derived phenotype–cell type relationships, with predictive performance reaching AUC ≈ 0.71 depending on the dataset. These findings suggest that transcriptional patterns captured in healthy single-cell atlases may contain informative signals about disease-relevant cellular contexts when the relevant cell populations are represented. At the same time, the results highlight the limitations of current reference resources and the need for continued efforts to improve single-cell atlases, phenotype–tissue mappings, and benchmarking datasets linking rare disease phenotypes to cellular contexts.

## Introduction

Rare diseases, despite their individual low prevalence, collectively affect millions worldwide and pose persistent challenges for both diagnosis and treatment. The majority of these conditions have a genetic basis, with mutations that disrupt molecular and cellular pathways, ultimately manifesting as distinct and often severe phenotypes. Experimental approaches such as CRISPR-Cas9 gene editing, animal models, patient-derived iPSCs, and *in vitro* functional assays have become indispensable tools for validating the pathogenicity of specific variants and establishing the molecular and cellular pathways disrupted [1]. These strategies enable direct interrogation of gene function and have substantially advanced our understanding of rare disease biology.

A central challenge, however, is determining the specific cellular contexts in which pathogenic variants exert their effects. Identifying the relevant cell populations is critical for interpreting disease mechanisms and for selecting appropriate experimental systems for downstream validation. Despite their utility, experimental approaches in rare disease research are often limited in their ability to systematically resolve the precise cell types affected in human disease. Many rely on model systems that only partially recapitulate human tissue architecture and cellular diversity, and are further constrained by high costs, long timelines, and limited scalability.

Recent advances in single-cell transcriptomics have provided an unprecedented opportunity to address this gap by enabling the systematic characterization of gene expression at cellular resolution across diverse human tissues. Large-scale efforts have generated comprehensive single-cell atlases that capture the transcriptional diversity of healthy organs, and similar datasets are increasingly available for common diseases. However, for rare diseases, direct transcriptomic profiling of affected tissues remains largely infeasible, as many conditions originate during development or manifest in childhood, and access to relevant tissues is therefore limited. Consequently, traditional disease–control single-cell studies are not practical for most rare conditions.

In this context, a growing set of computational approaches leveraging single-cell data from non-diseased human tissues has emerged as a complementary strategy to infer the cellular contexts in which disease genes may act. These approaches are primarily intended to provide a practical and scalable framework to prioritize candidate cell populations for downstream experimental validation, particularly in settings where direct experimental interrogation is not feasible. However, the field remains at an early stage of development. Current methods are constrained by the availability, completeness, and representativeness of reference datasets—often derived from adult tissues—and their performance, robustness, and domain of applicability are still being actively evaluated. In parallel, the biological assumptions underpinning these approaches, including the extent to which disease-relevant cellular states can be inferred from gene expression patterns in healthy tissues, remain incompletely understood.

Several studies implementing these approaches have shown that expression patterns of disease-associated genes in healthy single-cell datasets can highlight disease-relevant cell types in complex disorders such as schizophrenia [2], Parkinson’s disease [3], as well as in Mendelian conditions [4]. Most existing approaches rely on the assumption that disease-relevant cellular environments can be approximated by identifying cell types in which causal genes are preferentially expressed under physiological conditions. Although this principle has proven informative, it also presents important limitations. Many genes implicated in rare diseases are broadly expressed across tissues [5], which reduces the specificity of strategies based solely on expression enrichment and suggests that alternative ways of linking disease genes to cellular contexts may be required.

To overcome the limited specificity of approaches based solely on cell-type–specific expression, we previously proposed a framework based on a different biological premise: that genes contributing to the same phenotype tend to participate in shared regulatory programs and therefore show coordinated expression patterns within relevant cellular contexts [6]. Under this view, cell populations relevant to a phenotype can be identified by examining the co-expression relationships among phenotype-associated genes. Applying this strategy, we showed that prioritizing cell types based on gene co-expression improved the recovery of known gene–phenotype associations compared with approaches relying exclusively on expression specificity. However, implementing this idea required constructing cell-type–specific co-expression networks, which becomes computationally demanding as single-cell atlases continue to expand. Current initiatives are generating datasets containing millions of cells across multiple tissues and individuals, making it increasingly important to develop strategies that remain tractable at this scale while allowing cellular heterogeneity to be explored at finer resolution.

In the present study, we examine an alternative biological hypothesis for linking disease genes to cellular contexts. Rather than focusing on coordinated regulation among phenotype-associated genes, we ask whether cells that express a larger number of such genes than expected from their overall transcriptional activity may represent contexts particularly susceptible to disruption. To explore this idea, we analyze healthy human single-cell atlases together with curated gene–phenotype relationships, assessing whether this signal can be detected across a broad range of phenotypes and tissues. This formulation allows the hypothesis to be evaluated systematically at single-cell resolution while remaining computationally scalable for the analysis of large reference atlases.

## Results

### Biological grounding and overall approach

Our approach is based on a simple biological premise: we hypothetize that cells are more likely to be affected by a given phenotype if they express a larger number of genes associated with that phenotype compared with other cells in the same tissue.

Because the number of genes from any gene set detected in a cell is inherently influenced by its overall transcriptional activity, we first model the expected relationship between the total number of expressed genes and the number of genes associated with a given phenotype using linear regression. In this analysis, gene expression is treated as a binary variable: detected or not detected. A linear model was chosen for its simplicity and computational efficiency, and because visual inspection across phenotypes indicated that this relationship is well approximated by a linear trend in most cases. Importantly, this formulation proved broadly generalizable across the wide range of phenotypes analyzed, enabling a consistent framework for systematic evaluation of the method.

**Figure 1:**
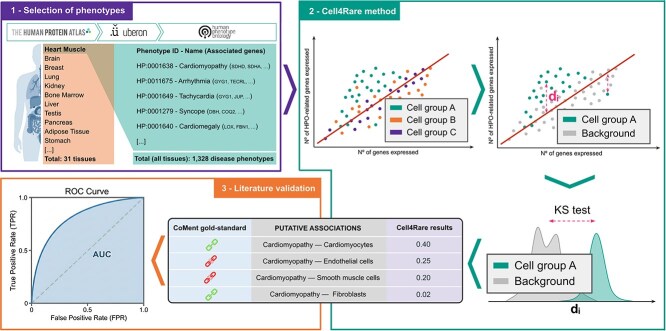
**Overall approach. (1) selection of phenotypes for analysis.** We obtained scRNA-seq data from non-diseased human tissues from the HPA and TS datasets and mapped each tissue to the corresponding phenotypes in the human phenotype ontology (HPO). **(2) Cell4Rare method.** For each tissue-phenotype pair, we applied Cell4Rare to compute associations with annotated cell groups (cell types and, in the HPA dataset, also cell clusters). **(3) literature validation.** We systematically evaluated the resulting phenotype-cell type associations using previously reported associations from the literature compiled with CoMent.

Each cell is then evaluated according to its deviation (*d_i_*) from the expected relationship (Fig. 1), allowing us to quantify whether it expresses more phenotype-associated genes than anticipated given its overall transcriptional profile. This deviation provides a score that can be used to prioritize individual cells for a given phenotype. To identify statistically significant patterns across predefined cell populations (e.g., cell types or clusters), we applied a signed two-sample Kolmogorov–Smirnov (KS) test, comparing whether the distribution of deviations within each population is greater to that observed across all other cells. This approach enables the identification of cell types or clusters containing cells that consistently co-express a higher number of phenotype-associated genes than the remaining cells.

To evaluate our approach, we analyzed two single-cell RNA sequencing (scRNA-seq) datasets from non-diseased human tissues compiled in the Human Protein Atlas (HPA) [7] and Tabula Sapiens (TS) [8]. We used the provided cell-type annotations for both datasets and, in the case of HPA, also incorporated cell cluster definitions to capture finer-grained transcriptional heterogeneity. For each tissue, we curated a set of relevant phenotypes from the Human Phenotype Ontology (HPO) [9]. Phenotypes were selected based on anatomical mappings provided by the HPO and further refined through manual curation, as described in the Methods section and Supplementary Material. For each phenotype, we compiled their associated genes from the HPO.

We then applied our framework, Cell4Rare, to identify cell types (and in the HPA dataset also clusters) for each phenotype. Finally, we systematically validated our predictions by comparing them to known phenotype–cell type associations automatically compiled from the literature. An overview of the analytical workflow is provided in Fig. 1 (see also the Methods section for additional details).

Here, we present the results derived from the HPA dataset. Analyses based on the TS dataset are provided in the Supplementary Material (Figs S1, S2 and Table S1).

### Inferred phenotype–cell type and cluster associations

We analyzed single-cell RNA-seq data spanning 31 human tissues curated by the HPA [7], aggregated from multiple independent studies [8,10–28]. The HPA dataset includes annotations for 557 distinct cell clusters, corresponding to 85 unique cell types. We mapped a total of 2630 phenotypes, including 1328 non-redundant phenotypes — defined as the most specific HPO terms meeting the gene count threshold described in the Methods. Some phenotypes were mapped to multiple tissues. Details regarding the number of phenotypes analyzed per tissue and the phenotype size distribution are provided in Fig. S3.

To quantify phenotype–cell set associations, we used the test statistic *D_n,m_*​ from the signed two-sample Kolmogorov–Smirnov (KS) test as a prediction score (see Methods). This statistic captures the divergence between two empirical distributions: one representing the deviation from regression in a given cell population (e.g., cell type or cluster), and the other representing the deviation from all remaining cells in the tissue. *D_n,m_*​ ranges from 0 to 1. Because we applied a signed version of the test, higher values of *D_n,m_*​ ​(closer to 1) reflect a higher number of phenotype-genes expressed for the cells of the population of interest compared to other cells in the tissue.

Using the HPA dataset, we analyzed 31 tissues and 2630 phenotypes, including 1328 non-redundant phenotypes, yielding a total of 27,603 phenotype–cell type pairs (13,918 corresponding to non-redundant phenotypes) (Table S2). Results from the TS dataset—covering 24 tissues and 2193 phenotypes, of which 1114 are non-redundant—are provided in Table S1 and Fig. S2.

To evaluate potential biases in our results, we assessed the correlation between phenotype size—defined as the number of genes associated with each phenotype—and *D_n,m_*​​ in the HPA dataset. The Pearson correlation coefficient was −0.016 (P = 5.43 × 10^−9^), indicating a negligible association despite the statistical significance due to large sample size. Similarly, the correlation between the number of cells within a cell type and *D_n,m_*​ yielded a Pearson coefficient of −0.091 (P = 1.44 × 10^−232^). These results suggest that *D_n,m_*​ is not biased by either phenotype size (number of genes) or cell type (number of cells).

**Figure 2:**
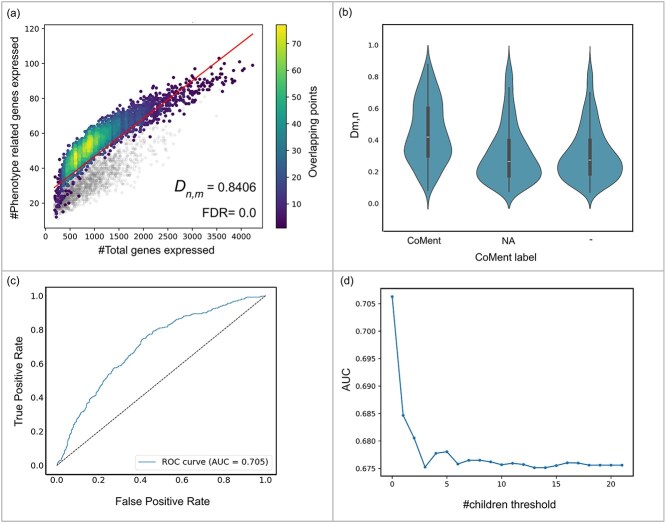
**Overview of results on HPA data.** (a) Results for dilated cardiomyopathy analysed using HPA heart muscle scRNA-seq data. Colored points represent cardiomyocytes, and grey points represent other cell types. (b) Distribution of the maximal D_n,m_ value per cell type grouped by literature co-mention. ‘CoMent’ indicates significant co-mention, ‘–’ indicates non-significant or absent co-mention, and ‘NA’ indicates no available information. (c) ROC curve and AUC value for the CoMent-based literature validation of results on HPA data, following the ‘Best cell type per phenotype’ criterion. (d) AUC values when upper-level phenotypes are included in the analysis.

As a representative example, our analysis of heart muscle scRNA-seq data revealed a strong and specific association between *dilated cardiomyopathy* (HP: 0001644) and cardiomyocytes (*D_n,m_*​ = 0.84, FDR = 0.0) (Fig. 2a). In contrast, substantially lower scores were observed for other cardiac cell types present in the dataset (Fig. S4), including endothelial cells (*D_n,m_*​ = 0.0, FDR = 1), smooth muscle cells (*D_n,m_*​ = 0.0004, FDR = 1) and fibroblasts (*D_n,m_*​ = 0.0005, FDR = 0.0). These results are consistent with the known pathophysiology of dilated cardiomyopathy, which is characterized by ventricular dilation and systolic dysfunction that often progresses to heart failure. Many of the genetic causes of this condition disrupt cardiomyocyte structure and contractility [29].

In the HPA dataset, certain cell types are represented by multiple cell clusters. These cases of multiple clusters may reflect transcriptionally distinct subtypes within a given cell type (e.g., different types of t-cells in PBMC and other tissues or functionally distinct neurons in the brain). To explore this additional layer of heterogeneity, we extended our analysis to infer associations between phenotypes and individual cell clusters. In total, we evaluated 86,756 phenotype–cell cluster pairs (Table S3).

Of particular interest are those cases where a particular cell cluster has a higher score than the corresponding cell type (Fig. S5, Table S4). Some of the most extreme examples include specific clusters of bronchus ciliated cells and bronchus club cells related to different respiratory pathologies like recurrent *pneumonia*, *bronchiectasis* and *cough*. Also, specific clusters of brain excitatory neurons related to neurological problems including *abnormal prosody*, *aplasia of the olfactory bulb* and *dementia*.

### Literature validation

To evaluate our results systematically in the absence of curated phenotype-cell type associations, we compiled a set of known phenotype–cell type relationships from the literature using CoMent [30], a method that quantifies the enrichment of term co-mentions in PubMed. Phenotype-cell types pairs significantly co-mentioned in the literature (CoMent *p* < 0.001) were considered positive relations, whereas all remaining pairs were treated as negative relations.

We assessed potential biases in the validation dataset by comparing the size distribution of phenotypes—defined by the number of associated genes—between those with at least one reported cell-type association in the literature and those without any such association. Non-redundant phenotypes with at least one co-mention exhibited a significantly greater number of associated genes (*p* = 1.906 × 10^−6^). In contrast, no significant differences were observed when the analysis was extended to redundant phenotypes or to the full phenotype set (non-redundant plus redundant) (*p* = 0.583 and *p* = 0.058, respectively).

We next assessed whether the lexical complexity of phenotype terms influences their likelihood of being represented in our validation dataset. Phenotypes with documented cell-type associations in the literature exhibited significantly lower lexical complexity, characterized by fewer words and fewer characters (signed Kolmogorov–Smirnov test, *p* = 0 for both comparisons).

To assess the performance of our approach, we compared the phenotype–cell type associations inferred with the positive relations identified by CoMent. *Receiver Operating Characteristic (ROC)* curves were generated using decreasing *D_n,m_* values as prediction thresholds, and performance was quantified using the *area under the curve* (AUC). For the HPA dataset, associations were computed both at the cell type and cell cluster levels; however, only cell type associations could be directly validated against the literature. Therefore, we evaluated performance using three alternative criteria: a) Best Cell Type, considering only scores calculated at the cell type level; b) Best Cell Cluster, considering the highest score among cell clusters within each cell types; and c) Best Cell Cluster or Cell Type, considering the highest score obtained at either level. For each phenotype, only a single positive relation was considered, regardless of the number of tissues involved (i.e., for phenotypes mapped to multiple tissues, only the highest *D_n,m_* value was retained). Alternative ROC curves and AUC values considering multiple tissues resulted in lower performances (Fig. S6).

The distribution of *D_n,m_* values per cell type (for Best Cell Type criterion), stratified by literature support (significant, non-significant, or unavailable), is shown in Fig. 2b.

The best performance was obtained using the Best Cell Type criterion, with an AUC = 0.71 (Fig. 2c). The distributions of *D_n,m_* values and ROC curves of the Best Cell-Cluster (AUC = 0.66) and Best Cell Cluster or Cell Type (AUC = 0.69) criteria, are presented in Figs S6 and S7.

We also evaluated the performance when more general phenotypes were considered according to the HPO hierarchical structure. As shown in Fig. 2d (for Best Cell Type criterion), the highest performance was obtained when only non-redundant (i.e., most specific) phenotypes were included. Allowing more general phenotypes —those with a larger number of child terms within the analyzed set—resulted in progressively lower AUC values.

It is also important to note that phenotype–cell associations predicted by our method but not significantly co-mentioned in the literature may nevertheless be biologically supported. For example, we identified an association between the phenotype *Productive cough* and ciliary cells in both bronchus (*D_n,m_* = 0.87, FDR = 0) and lung (*D_n,m_* = 0.67, FDR = 4.1 × 10^−146^). Although this relationship was not detected as a significant co-mention by CoMent, it is consistent with known biological mechanisms. Impaired ciliary function disrupts mucus clearance in the airways, which frequently leads to chronic productive cough [31].

In addition, several associations not supported by significant co-mentions in our validation set involve cell types that are inherently linked to the definition of the phenotype. Notably, multiple phenotypes showed high association scores with skeletal myocytes (muscle fibers) in muscle tissue. These include *myofibrillar myopathy* (*D_n,m_* = 0.89, FDR = 8.8 × 10^−198^), which reflects intrinsic pathology of the contractile apparatus within skeletal muscle fibers; *abnormal sarcomere morphology* (*D_n,m_* = 0.88, FDR = 1.4 × 10^−193^), referring to structural abnormalities of the sarcomere—the fundamental contractile unit of skeletal myocytes; and *fatty replacement of skeletal muscle* (*D_n,m_* = 0.86, FDR = 1.5 × 10^−186^), defined by degeneration of muscle fibers with subsequent adipose tissue infiltration.

Comparable examples were observed in other tissues. These include *abnormal cardiomyocyte morphology* associated with cardiomyocytes (*D_n,m_* = 0.76, FDR = 0), *cone/cone-rod dystrophy* associated with cone photoreceptor cells (*D_n,m_* = 0.71, FDR = 9.7 × 10^−58^), and *respiratory insufficiency due to defective ciliary clearance* associated with ciliated cells (*D_n,m_* = 0.67, FDR = 5.9 × 10^−146^). All of these represent bona fide biological associations that were incorrectly labeled as negatives in the validation set, either because co-mentions did not reach statistical significance or because no instances of the phenotype were identified in the literature.

The performance for the TS dataset, AUC = 0.62 (Fig. S2), is lower than that obtained for the HPA dataset (AUC = 0.71).

### Differential expression-based baseline

To compare our method against a baseline approach, we performed differential expression analysis on the nTPM (normalized Transcripts Per Million) single-cell HPA dataset as in [6]. For each tissue and cluster, we computed gene-wise fold changes (logFC) by comparing expression in the target cluster to the mean across remaining clusters within the same tissue. For each phenotype–cluster pair, we tested whether phenotype-associated genes exhibited higher logFC values than non-associated genes using the signed two-sample Kolmogorov–Smirnov statistic *D_n,m_*​ as a prediction score. This analysis was restricted to cell clusters, as cell type–resolved nTPM values are not available in HPA.

We applied the same literature-based validation framework described above. Best performance was obtained using the Best Cell-Cluster (global) criterion—selecting, for each phenotype, the top-scoring cluster across all tissues—yielding an AUC of 0.52, lower than Cell4Rare (AUC = 0.66). AUC values for alternative criteria are provided in Fig. S8. Our method consistently outperformed the differential expression–based baseline across all criteria.

## Discussion

In this study, we examined the extent to which single-cell gene expression data from healthy human reference atlases can be used to identify cell populations potentially affected in genetic diseases sharing specific phenotypic manifestations using a novel computational framework. Our analysis focused on disorders with known causal genes—many of which correspond to rare diseases—where genetic variants exert strong and well-characterized effects on phenotype. This setting provides a tractable framework for investigating how disruptions in individual genes may translate into cellular dysfunction and clinical manifestations. Two considerations motivated this approach. First, the study of disease mechanisms in rare disorders is frequently constrained by the limited availability of patient-derived tissues, particularly for congenital and pediatric conditions that constitute the majority of rare diseases. This is not only due to technological or economical constraints but in many cases to ethical issues as well, and hence it is not expected to change in the future. Second, the analysis explores a biological premise in which cells that express a larger number of phenotype-associated genes than expected from their overall transcriptional activity may represent contexts particularly susceptible to disruption. Under this view, identifying such cells within healthy single-cell atlases can provide clues about the cellular environments in which disease genes are most likely to exert their effects. More broadly, linking phenotype-associated genes to cellular contexts may also help clarify the cellular basis of related phenotypic manifestations observed in more common, multifactorial diseases, where the underlying genetic architecture is often more complex.

Compared with previous strategies that infer disease-relevant cell types from non-diseased single-cell data, this study evaluates an alternative conceptual framework for connecting disease genes with cellular contexts. Earlier approaches relied primarily on the cell specificity of disease genes or on co-expression patterns among them. In contrast, the hypothesis examined here focuses on the collective presence of phenotype-associated genes within individual cells, asking whether cells expressing an unexpectedly large number of such genes may represent vulnerable cellular contexts. Implementing this idea in a simple statistical formulation makes it possible to evaluate the hypothesis across large single-cell atlases while remaining computationally tractable. In addition, by operating at single-cell resolution, the analysis allows signals to emerge from heterogeneous cellular populations rather than relying exclusively on predefined cell types, although the systematic validation is ultimately performed at the level of annotated cell types.

Applying this framework across a large collection of phenotype–gene associations revealed detectable signals linking disease phenotypes to specific cellular contexts. Although the best predictive performance was moderate (AUC ≈ 0.71), this result was obtained despite several sources of biological and technical mismatch between the reference data and the conditions being modeled. In particular, the single-cell atlases used in this study are derived primarily from healthy adult tissues, whereas many of the analyzed phenotypes arise during development or childhood and may involve cellular states that are not represented in these references. In addition, phenotype annotations and literature-derived cell type associations used for evaluation are inherently incomplete and heterogeneous. Together, these factors likely impose an upper bound on the level of concordance that can be expected when evaluating the hypothesis using currently available resources. Nevertheless, the presence of detectable signals suggests that transcriptional patterns captured in healthy single-cell atlases retain information about the cellular contexts in which disease-associated genes may act, allowing candidate phenotype-relevant cellular populations to be prioritized even when direct disease-state data are unavailable.

The primary goal of this study was to identify the cell populations within a given tissue that are most relevant to the manifestation of a phenotype. However, certain phenotypes are mapped in the Human Phenotype Ontology (HPO) to multiple tissues, allowing to highlight differences in the relative strength of associations across biological systems. The statistic *D_n,m_*​ estimates the extent to which specific cell types preferentially express phenotype-associated genes. For example, *facial diplegia*, characterized by bilateral paralysis or weakness of the facial muscles, is classified in the HPO as both a nervous system and musculoskeletal abnormality. In our analysis, genes associated with this phenotype showed a strong association with skeletal myocytes in skeletal muscle tissue (*D_n,m_*​​ = 0.94) compared to other skeletal muscle cell types, whereas the strongest association in the brain was observed for inhibitory neurons (cluster 30), with a substantially lower score (*D_n,m_*​​ = 0.27). Similarly, *anorexia*, mapped to both the nervous and digestive systems, showed stronger associations with immune cells in digestive tissues (colon, stomach, rectum, and small intestine; *D_n,m_*​ ​values ranging from 0.40 to 0.70), than with microglial cells, the highest scoring in the brain (*D_n,m_*​ = 0.28).

These examples illustrate how the framework can help highlight potentially distinct cell populations underlying shared phenotypic manifestations in different tissues. Importantly, stronger associations in one tissue do not exclude the possibility that the phenotype might result from disruptions in multiple systems. *Difficulty walking* provides a representative example. Among the two tissues to which this phenotype is mapped in the HPO, the highest score was observed in skeletal myocytes from skeletal muscle (*D_n,m_*​ = 0.87; 133 phenotype-associated genes expressed), followed by neuronal cells in the brain (*D_n,m_*​ =0.51; 223 associated genes). These results suggest that this phenotype may arise through at least two distinct cellular routes: impairment of skeletal muscle cells affecting motor function, or dysfunction of neuronal populations involved in movement control. The larger number of phenotype-associated genes expressed in neuronal cells—many of which are not detected in skeletal muscle—supports the idea that different sets of genes may contribute to mechanistically distinct forms of the same clinical manifestation.

### Study limitations

Despite these strengths, our study has several limitations arising from both the underlying data and the evaluation strategy. Gene–phenotype associations in the HPO are primarily derived from gene–disease relationships, which are causal to the disease but do not always reflect cell-autonomous mechanisms. In some cases, annotated phenotypes may represent secondary consequences of a disease rather than direct outcomes of the underlying genetic perturbation. Although such distinctions are not explicitly captured in the ontology, our statistical framework appears sufficiently robust to tolerate this level of noise while still identifying meaningful patterns.

The use of single-cell data derived from adult tissues represents another important limitation. Many phenotypes analyzed in this study may arise during development or involve cellular states that are absent from adult reference atlases. General low scores for all cell types in a tissue may indicate that relevant cell populations are not adequately represented in the dataset. For instance, *frontal bossing*, analyzed in brain tissue, likely reflects developmental or skeletal processes that would be better captured in embryonic or craniofacial tissues. Similarly, phenotypes such as *synophrys*, *highly arched eyebrows*, and *low anterior hairline*, analyzed in skin tissue, may depend on structures such as hair follicles that are underrepresented in the adult samples analyzed. Hematological phenotypes such as *myelodysplasia* or *acute myeloid leukemia* may involve rare or dynamic hematopoietic populations not well captured in healthy adult bone marrow datasets.

The quality and granularity of cell type annotations also strongly influence the results. To highlight this, we compared results obtained from six tissues (lung, prostate, salivary gland, thymus, tongue and vascular), corresponding to the same scRNAseq data (TS), but annotated independently by the Human Protein Atlas (HPA) or Tabula Sapiens (TS). The highest *D_n,m_*​ values for phenotypes vary substantially depending on the dataset (Fig. S9).

Differences in cell composition captured by distinct experiments on the same tissue can also influence the outcome. For example, the TS skin dataset contains very few epithelial cells (1.1%) and lacks keratinocytes, whereas the HPA skin dataset includes basal and suprabasal keratinocytes representing approximately 15% of the cells. Because keratinocytes are central to skin physiology, analyses of skin phenotypes using the TS dataset are expected to yield less reliable results simply due to limitations in cell-type representation.

Additional limitations arise from the validation strategy. Our evaluation relies primarily on literature-derived phenotype–cell type associations obtained through co-mentions in the biomedical literature, which may not imply causal relations. Although the scientific literature provides the most comprehensive aggregation of current biological knowledge, such associations are inherently noisy, incomplete, and biased toward well-studied phenotypes and cell types. For instance, polysemous terms can generate erroneous matches (e.g., *irritability* as an emotional state may be confused with physiological irritability, such as in *bowel irritability*), genuine associations may be overlooked if they appear in only a small number of publications, and co-mention does not necessarily imply a direct mechanistic relationship. In addition, some phenotypes and cell annotations could not be found in the literature, due to their lexical complexity.

Challenges also arise in mapping ontology terms used for validation. Some HPA cell annotations, including *mixed immune cells*, *undifferentiated cells*, and *mixed cells,* cannot be reliably mapped to specific Cell Ontology terms*.* While such categories may still capture biologically relevant signals, —for example, high scores for *adenocarcinoma of the colon* (*D_n,m_*​ = 0.65) and *recurrent infection of the gastrointestinal tract* (*D_n,m_*​ = 0.62) in mixed immune cells—they must be treated as false positives during validation due to the lack of precise ontology alignment. Similarly, mappings from HPO phenotypes to tissues can occasionally produce implausible associations, such as *male infertility* being mapped to the fallopian tube and endometrium tissues, which yielded high scores for ciliated cells.

Ideally, predicted associations would be evaluated using disease-state single-cell datasets or systematic perturbation experiments. However, disease-state resources remain limited, particularly for rare diseases and developmental phenotypes in humans. Experimental perturbation models can provide complementary evidence, but they are often performed in non-human systems and frequently involve strong knockouts or knockdowns that may not accurately reflect the partial or regulatory genetic perturbations observed in human rare diseases. Under these constraints, literature-based concordance provides a pragmatic—albeit imperfect—benchmark for assessing biological plausibility. Despite its limitations, CoMent currently represents one of the most comprehensive available resources for systematically linking disease phenotypes to candidate cell types in the absence of curated gold-standard datasets.

### Conclusion

Taken together, our results highlight both the potential and the current limitations of leveraging healthy single-cell reference data to infer cellular contexts for genetic disease phenotypes. Although our approach outperforms a differential expression–based baseline, the overall predictive accuracy remains modest, underscoring the intrinsic difficulty of linking phenotype-associated genes to specific cellular states using reference atlases alone.

These limitations arise from several structural constraints. Phenotype–tissue mappings are often incomplete and defined at a coarse tissue level, lacking the cellular and contextual resolution required for integration with single-cell data. In addition, current reference atlases are largely restricted to adult samples and fail to capture developmental stages relevant to many congenital and pediatric-onset diseases. The limited representation of rare or transient cell states further constrains the ability to resolve disease-relevant cellular contexts.

Progress is also impeded by the absence of comprehensive, high-quality resources systematically cataloguing validated associations between rare diseases and cell types, as well as between phenotypes and cellular contexts. The scarcity and fragmentation of such datasets limit both method development and the ability to perform rigorous, standardized benchmarking.

Addressing these limitations will require tighter integration of genetic, phenotypic, and single-cell data, together with the development of curated, community-driven resources linking diseases and phenotypes to specific cell types. Expanding reference atlases to better capture cellular diversity, developmental trajectories, and context-specific states will be essential to improve the interpretability and accuracy of computational approaches.

## Methods

### Single-cell expression data

We obtained single-cell transcriptomic data (scRNA-seq) from non-diseased human tissues from two sources: (a) 31 tissues from the Human Protein Atlas (HPA) (https://v23.proteinatlas.org/; [7]; version 23.0) and (b) 24 tissues from Tabula Sapiens (TS) (https://tabula-sapiens.sf.czbiohub.org/ [8]; 2022 version). For each tissue, we also retrieved the corresponding cluster and cell-type annotations provided by the respective datasets.

Disease phenotypes and their associated gene annotations were obtained from the Human Phenotype Ontology (HPO) (https://hpo.jax.org/, [9]; v2024-01-16). Anatomical entities were retrieved from the Uberon Multi-species Anatomy ontology (UBERON) (https://www.ebi.ac.uk/ols4/ontologies/uberon; [32]; v2024-01-27).

### Tissue to phenotype mapping

To establish a compendium of disease phenotypes associated with each tissue, we combined two complementary approaches: (a) automated mapping based on UBERON relations and (b) targeted manual curation. For the UBERON-based mapping (a), each tissue was annotated with its corresponding UBERON term and expanded to include all descendant terms. We then retrieved associated Human Phenotype Ontology (HPO) terms using the HPO .owl file and further expanded these to include all descendant phenotypes.

Manual curation (b) was introduced to complement the limited coverage of ontology-based mappings. Rather than performing exhaustive curation, we selected a set of high-level phenotype terms with clear tissue associations (Table S5) and propagated these mappings to their descendant terms in the HPO hierarchy. The criteria to select these high-level phenotype terms is detailed in Supplementary Material. This strategy increases coverage but may introduce coarse or biologically imprecise associations due to the hierarchical structure of HPO, particularly in cases where phenotype branches diverge into context-specific subcategories (e.g., sex-specific phenotypes).

The final tissue–HPO mappings were obtained by combining both approaches. This procedure was performed independently for HPA and TS tissues.

For each selected phenotype, we retrieved the associated gene sets from the HPO database, which aggregates gene–disease associations derived from OMIM [33] and Orphanet [34]. Specifically, we obtained the list of genes related to each phenotype from the HPO annotation file *phenotype_to_genes.txt*. For each tissue–phenotype pair, we computed the maximum number of phenotype-associated genes expressed by any individual cell in the corresponding scRNA-seq dataset, considering a gene as expressed if its transcript count exceeded zero. Phenotypes for which this maximum number of expressed genes was fewer than ten were excluded, as such sparse representation would likely yield noisy results in subsequent analyses.

Finally, for each tissue, we selected only the most specific, non-redundant phenotypes from the resulting list—i.e., those phenotypes that have no children among the set of phenotype terms associated with that tissue. This constituted the final set of phenotypes used in downstream analyses.

### Expression analysis

We used the scRNA-seq binarized transcript counts to determine which individual cells within a tissue expressed more genes related to a given phenotype than expected based on their total number of expressed genes. For each phenotype associated with a tissue, we constructed a two-dimensional space in which each cell was represented by two coordinates: *x,* the total number of genes expressed by the cell, and *y,* the number of phenotype-related genes expressed by the cell. A gene was considered expressed if its transcript count was greater than zero.

We then performed an ordinary least squares linear regression on this set of points. Cells located above the regression line were interpreted as expressing more phenotype-related genes than expected. For each cell, we computed *d_i_*, the difference between the observed number of phenotype-related genes *y_i_* and the expected value predicted by the linear model. This yielded a distribution of distances to the regression line for all cells in the tissue.

No explicit batch correction was applied, as all single-cell data were generated from the same tissue using consistent laboratory protocols within the same resource, thereby minimizing major sources of technical variability. Gene expression was binarized (expressed versus not expressed) to reduce sensitivity to sequencing depth and other amplitude-related effects. The analysis accounts for variability in gene detection by evaluating whether the number of expressed phenotype-associated genes exceeds expectations given the total number of genes detected in each cell.

### Statistical assessment

We performed a signed two sample Kolmogorov–Smirnov test (KS) for each cell group (cluster or cell type) to assess whether the distribution of distances to regression (*d_i_*) within that group was shifted toward higher values compared to the distribution in the remaining cells. Resulting p-values were corrected for multiple testing using the Benjamini-Hochberg false discovery rate (FDR). We used the KS test statistic (*D_n,m_*​) as a score to quantify the strength of the association. This statistic measures the maximum difference between the empirical cumulative distribution functions of the two samples: cells within the group and all remaining cells. The KS statistic ranges from 0 to 1, with higher values indicating a greater separation between the two distributions. Formally, *D_n,m_*​ is defined as:


$$ Dn,m=\mathit{\sup}\mid Fn(x)- Gm(x)\mid x $$

where F*_n_*(x) and G*_m_*(x) denote the empirical cumulative distribution functions of the two samples, and *sup* represents the supremum function, corresponding to the maximum vertical distance between the two distributions.

### Literature validation

We compiled a set of known phenotype-cell type associations from the literature using CoMent [30], a tool that identifies statistically significant co-mentions of biomedical concepts across abstracts in the entire PubMed corpus. A detailed description of the method is provided in [35]. To align our results with the associations compiled by CoMent, each cell type annotation in the scRNA-seq datasets was mapped to the corresponding Cell Ontology (CL) term [36] term. In cases where anatomical entities are primarily defined by their constituent cell types, we also included the corresponding Uberon terms (e. g. mapping the HPA annotation ‘endothelial cell’ to the Uberon term ‘Endotelium’). Cell clusters were mapped indirectly through their associated cell type annotations.

Overall, 81 out of 85 HPA cell types were successfully mapped to CL and/or Uberon terms (Table S6). The unmapped annotations were ‘excitatory neuron’, ‘mixed cell types’, ‘mixed immune cell’, and ‘undifferentiated cell’. In the Tabula Sapiens (TS) dataset, CL annotations were already available; therefore, all 177 cell types were directly mapped to CL identifiers.

We compiled all known associations between HPO and CL terms, as well as between HPO and Uberon terms, with CoMent *p-value* < 0.001. Associations with *p-value* ≥ 0.001 (denoted as ‘-’ in the results) and HPO terms that were never co-mentioned with any cell type (denoted as ‘NA’) were treated as negative relations.

Using this set as a reference, we assessed the performance of our method. Receiving Operator Characteristic (ROC) curves were generated using decreasing *D_n,m_*​ values as prediction thresholds, and performance was quantified by calculating the Area Under the Curve (AUC).

For the HPA dataset, cell annotations are available at both the cell type and cell cluster levels, whereas literature-based validation is limited to cell types. To account for this difference, we defined three alternative evaluation criteria for this dataset: (a) considering only scores calculated at the cell type level; (b) considering the highest score among cell clusters within each cell type; and (c) considering the highest score obtained at either level. For each phenotype, only a single positive association was retained regardless of the number of tissues involved. Specifically, when a phenotype was mapped to multiple tissues, only the highest *D_n,m_* value across tissues was considered.

### Differential expression baseline method

We implemented a differential expression analysis [6] as a baseline for comparison. nTPM values for each gene and cluster were obtained from the HPA dataset. For each cluster, we computed the fold change (logFC) as:


$$ logFC=\mathit{\log}\left(\frac{TPM_{cluster}}{TPM_{background}}\right) $$

where TPM*_cluster_* denotes the nTPM value of a given gene in the target cluster and TPM*_background_*​ corresponds to the mean nTPM value of that gene across the remaining clusters within the same tissue. To reduce technical noise, values of nTPM lower than 1 were thresholded to 0 (i.e., treated as no expression). To avoid numerical instability due to division by zero or undefined logarithms, a small pseudocount was added to all nTPM values (we evaluated +0.1, +0.01, and + 0.001).

To assess the association between tissue-related phenotypes and clusters, we applied a signed two-sample Kolmogorov–Smirnov test, comparing the distribution of logFC values for phenotype-associated genes against that of background genes within the same cluster. The test statistic *D_n,m_*​ was used as a quantitative measure of the strength of association between each phenotype and cluster.

We performed literature-based validation of the differential expression results following the methodology described in the previous section. This validation was restricted to the Best Cell-Cluster criterion, as cell type–resolved nTPM values for individual tissues are not available in HPA version 23.0.

**Key Points**

- Identifying which cell populations are affected by disease genes remains a major challenge in rare disease research, particularly because patient-derived tissues are often inaccessible.
- Reference human single-cell atlases obtained from healthy tissues offer an opportunity to explore disease-relevant cellular contexts when direct patient data are unavailable.
- We test the hypothesis that cells expressing more genes associated with a phenotype than expected from their overall transcriptional activity may represent cellular contexts particularly susceptible to disruption.
- Evaluating this idea across more than 1300 phenotypes and over 30 tissues reveals partial concordance with literature-derived phenotype–cell type relationships, with moderate but detectable predictive signal.
- These results suggest that healthy single-cell reference atlases may contain informative signals about disease-relevant cellular contexts while highlighting the need for improved mapping of reference datasets and benchmarking resources.

## Supplementary Material

Supplementary_material_bbag426

## Data Availability

Data analysed in this work is available at: HPA data: https://v23.proteinatlas.org TS data: https://tabula-sapiens.sf.czbiohub.org HPO data: https://hpo.jax.org UBERON data: https://www.ebi.ac.uk/ols4/ontologies/uberon CoMent: https://csbg.cnb.csic.es/coment2 And supplementary tables.

## Code availability

All the code generated in this work is available at: https://github.com/JNovoaR/Cell4Rare
