# Supplementary material for: Exploring cell-type associations of rare disease phenotypes using non-diseased cell atlases
Source: Brief Bioinform. 2026 Aug 6;27(4):bbag426. doi: 10.1093/bib/bbag426 (PMC13446510; doi:10.1093/bib/bbag426)
Supplement: Supplementary file 1 [file supplementary_material_bbag426.zip › Supplementary.docx]

**Supplementary material - Novoa *et al*. “Discovering cell types underlying rare disease phenotypes using scRNA-seq data from non-diseased tissues”**

**Criteria for the manual annotation of phenotypes (HPO terms) to HPA or TS tissues**

To perform the manual mapping (b), we aimed to identify one or more broad HPO terms referring to abnormalities in the morphology and/or physiology of each tissue. We followed these criteria:

1. **Direct tissue-specific terms:** If available, we selected HPO terms explicitly referring to abnormalities of the given tissue (or one of its exact synonyms) using the format “Abnormality of the {tissue},” including both morphological and physiological abnormalities among their descendants (e.g., “Abnormality of the breast” for the tissues “Breast” [HPA] and “Mammary” [TS]).
2. **Separate morphological and physiological terms:** If a combined term was not available, we identified a pair of HPO terms separately covering morphological and physiological abnormalities of the tissue (or its synonyms).
3. **Broader anatomical terms:** If tissue-specific terms were unavailable, we selected HPO terms describing morphological and physiological abnormalities of broader anatomical entities that fully encompass the tissue.
4. **Morphological plus related physiological term:** If no broader physiological term was available, we selected a morphological HPO term as described above, together with a physiological term describing a biological function directly or partly performed by the tissue.
5. **Morphological term only:** If no clear physiological function could be ascribed to the tissue, we annotated it with the morphological HPO term alone (e.g., “Abnormality of the thymus”).
6. **Medical test results:** When available, we added HPO terms referring to abnormalities detectable in medical tests performed on the tissue (e.g., cardiac or pulmonary tests).

Finally, we expanded the manual annotation by including all descendant terms of the selected HPO terms. A preliminary annotation was constructed by combining this manual mapping with the automated HPO-based annotation.

**TABULA SAPIENS SUPPLEMENTARY FIGURES:**

**Supplementary Figure S1**. **Distribution of phenotype sizes (left) and number of phenotypes per tissue (right) in the Tabula Sapiens (TS) dataset**. Phenotype size is defined as the number of HPO-associated genes expressed in at least one cell of the mapped tissue. Phenotypes are colored by specificity: **non-redundant** phenotypes have no child terms among those mapped to the same tissue, while **redundant** phenotypes have at least one child term. Some phenotypes are mapped to multiple tissues; the table shows both the total number of phenotype–tissue pairs (allowing repeats) and the total number of unique phenotypes.

**Supplementary Figure S2. CoMent literature validation of the results of Cell4Rare applied over TS dataset**. Positive results were selected using two criteria: **(a, c)** the best cell type per phenotype–tissue pair (allowing multiple positives for phenotypes mapped to multiple tissues), and **(b, d)** the best cell type per phenotype (single positive per phenotype). Selected results were compared to phenotype–cell type pairs frequently co-mentioned in PubMed abstracts: **“CoMent”** indicates significant co-mention, **“–”** indicates non-significant or absent co-mention, and **“NA”** indicates no available information. Pairs tend to have higher D*_n,m_* values, especially under the global criterion. ROC curves and AUC values for both selection criteria are shown in (c) and (d). Performance was more modest than that observed using the Human Protein Atlas dataset.

**HUMAN PROTEIN ATLAS SUPPLEMENTARY FIGURES:**

**Supplementary Figure S3. Distribution of phenotype sizes (left) and number of phenotypes per tissue (right) in the Human Protein Atlas (HPA) dataset**. Phenotype size is defined as the number of HPO-associated genes expressed in at least one cell of the mapped tissue. Phenotypes are colored by specificity: **non-redundant** phenotypes have no child terms among those mapped to the same tissue, while **redundant** phenotypes have at least one child term. Some phenotypes are mapped to multiple tissues; the table shows both the total number of phenotype–tissue pairs (allowing repeats) and the total number of unique phenotypes.

**Supplementary Figure S4.** **Cell4Rare results for dilated cardiomyopathy using HPA heart muscle scRNA-seq data**. **(a)** Cardiomyocytes show a significant association with dilated cardiomyopathy (FDR < 0.001), whereas **(b–d)** endothelial cells, smooth muscle cells, and fibroblasts show no significant association. Dilated cardiomyopathy is characterized by ventricular dilation and systolic dysfunction, often progressing to heart failure, and arises from diverse genetic etiologies, many of which impair cardiomyocyte structure and contractility.

**Supplementary Figure S5. Comparison of D*_n,m_* values for the best cell-type versus the best cell cluster per phenotype in HPA.** Each point represents a phenotype mapped to an HPA tissue, with x as the D*_n,m_* value of the best cell type and y as that of the best cell cluster. Black line: x=y. Phenotypes where the best cell type and the best cell cluster are identical (i.e., the cell type consists of a single cluster) were excluded for clarity.

**Supplementary Figure S6**. **ROC curves and AUC values for CoMent literature validation of Cell4Rare results on the HPA dataset**. Positive results were defined as the highest-scoring (*D_n,m_*) cell-type association for each phenotype. Selecting the best-scoring association involves two considerations: (1) choosing the top cell type, the cell type containing the top-scoring cluster, or the best between both; and (2) for phenotypes mapped to multiple tissues, selecting the best per tissue or the overall best across tissues. ROC analyses were performed across six combinations of these criteria: **(a)** Best cluster per phenotype (tissue-specific), **(b)** Best cell type per phenotype (tissue-specific), **(c)** Best cluster or cell type per phenotype (tissue-specific), **(d)** Best cluster per phenotype (global), **(e)** Best cell type per phenotype (global), and **(f)** Best cluster or cell type per phenotype (global). Global and Best Cell-Type criteria consistently performed best, with peak performance achieved using the Best Cell-Type per phenotype (global) criterion (AUC = 0.705).

**Supplementary Figure S7**. **Distribution of Cell4Rare D*_n,m_* values (HPA dataset) for phenotype–cell type pairs supported or not supported by CoMent**. The gold standard consists of phenotype–cell type pairs frequently co-mentioned in PubMed abstracts: **“CoMent”** indicates significant co-mention, **“–”** indicates non-significant or absent co-mention, and **“NA”** indicates no available information. For Cell4Rare results, only the highest-scoring D*_n,m_* cell-type association per phenotype was selected. Selection involves two considerations: (1) choosing the top cell type, the cell type containing the top cluster, or the best between both; and (2) for phenotypes mapped to multiple tissues, selecting the best per tissue or the overall best across tissues. ROC analyses were performed across six combinations: **(a)** Best cluster per phenotype (tissue-specific), **(b)** Best cell type per phenotype (tissue-specific), **(c)** Best cluster or cell type per phenotype (tissue-specific), **(d)** Best cluster per phenotype (global), **(e)** Best cell type per phenotype (global), and **(f)** Best cluster or cell type per phenotype (global). Higher D*_n,m_* values are observed for pairs supported by CoMent, particularly under global criteria.

**Supplementary Figure S9. CoMent literature validation of the differential expressión baseline analysis performed over HPA cell-cluster data**. (a) AUC values for CoMent validations performed over Best Cell-Cluster HPA baseline analysis. This analysis was benchmarked with and without technical noise correction (nTMP <1 equaled to 0) and with different non-zero correction factors (small fixed values added to every nTPM to avoid mathematical indetermination due to nTPM=0). (b) ROC curve and AUC value for the best performing baseline analysis (Best Cell-Cluster HPA, global with technical noise correction factor and 0 0.1 non-zero correction factor) (c) Violin plot of the best performing baseline analysis. The gold standard consists of phenotype–cell type pairs frequently co-mentioned in PubMed abstracts: **“CoMent”** indicates significant co-mention, **“–”** indicates non-significant or absent co-mention, and **“NA”** indicates no available information. (d) Comparison of *D_n,m_*  values of best performing baseline analysis and correspondent Cell4Rare analysis (HPA Best Cell-Cluster, global). Each point represents a phenotype mapped to an HPA tissue. **Black line:** x=y.

**Supplementary Figure S8. Impact of cell-type annotation on Cell4Rare results**. Comparison of *D_n,m_*  values for the best cell type per non-redundant phenotype between HPA and TS for the six HPA tissues derived from TS. The underlying scRNA-seq data are identical, but cell annotations differ. Results show poor correlation in most cases, highlighting the influence of cell-type annotation on Cell4Rare outcomes. **Black line:** x=y.

**Supplementary Table S1. Cell4Rare statistical assessment of phenotype and TS Cell-Type pairs.** Columns from left to right: (1) Tissue, (2) HPO id, (3) HPO name, (4) Number of phenotype related genes (expressed at least once in the tissue), (5) Grouping level (in this case, Cell-Type), (6) Group name, (7) Number of cells belonging to the Cell-Type, (8) Number of cells in background (not belonging to the group), (9) *D_n,m_*  value of the signed Kolmogorov-Smirnov assessment between the Cell-Type and background group, (10) p-value of the test, (11) FDR correction (multiple testing Benjamini-Hochberg false discovery rate correction), (12) CoMent match (“CoMent” indicates significant co-mention, “–” indicates non-significant or absent co-mention, and “NA” indicates no available information) and (13) HPO specificity (Number of children phenotypes inside the ones mapped to the same tissue).

**Supplementary Table S2. Cell4Rare statistical assessment of phenotype and HPA Cell-Type pairs.** Columns from left to right: (1) Tissue, (2) HPO id, (3) HPO name, (4) Number of phenotype related genes (expressed at least once in the tissue), (5) Grouping level (in this case, Cell-Type), (6) Group name, (7) Number of cells belonging to the Cell-Type, (8) Number of cells in background (not belonging to the group), (9) *D_n,m_*  value of the signed Kolmogorov-Smirnov assessment between the Cell-Type and background group, (10) p-value of the test, (11) FDR correction (multiple testing Benjamini-Hochberg false discovery rate correction), (12) CoMent match (“CoMent” indicates significant co-mention, “–” indicates non-significant or absent co-mention, and “NA” indicates no available information) and (13) HPO specificity (Number of children phenotypes inside the ones mapped to the same tissue).

**Supplementary Table S3. Cell4Rare statistical assessment of phenotype and HPA Cell-Cluster pairs.** Columns from left to right: (1) Tissue, (2) HPO id, (3) HPO name, (4) Number of phenotype related genes (expressed at least once in the tissue), (5) Grouping level (in this case, Cell-Cluster), (6) Group name, (7) Number of cells belonging to the Cell-Cluster, (8) Number of cells in background (not belonging to the group), (9) *D_n,m_*  value of the signed Kolmogorov-Smirnov assessment between the Cell-Cluster and background group, (10) p-value of the test, (11) FDR correction (multiple testing Benjamini-Hochberg false discovery rate correction), (12) CoMent match (“CoMent” indicates significant co-mention, “–” indicates non-significant or absent co-mention, and “NA” indicates no available information) and (13) HPO specificity (Number of children phenotypes inside the ones mapped to the same tissue).

**Supplementary Table S4. Cell4Rare HPA statistical assessment of phenotype-Cell Clusters pairs with higher *D_n,m_*  value than the correspòndant phenotype-Cell Type pair.** Columns from left to right: (1) Tissue, (2) HPO id, (3) HPO name, (4) Number of phenotype related genes (expressed at least once in the tissue), (5) Grouping level (in this case, Cell-Cluster), (6) Group name, (7) Number of cells belonging to the Cell-Cluster, (8) Number of cells in background (not belonging to the group), (9) *D_n,m_*  value of the signed Kolmogorov-Smirnov assessment between the Cell-Cluster and background group, (10) p-value of the test, (11) FDR correction (multiple testing Benjamini-Hochberg false discovery rate correction), (12) CoMent match (“CoMent” indicates significant co-mention, “–” indicates non-significant or absent co-mention, and “NA” indicates no available information) and (13) HPO specificity (Number of children phenotypes inside the ones mapped to the same tissue).

**Supplementary Table S5. Manual mapping of HPA and TS tissues to HPO broad terms.** To select this manually curated broad phenotype terms we followed the criteria described in Supplementary Material. For some tissues no manual mapping was found. Other tissues were mapped to multiple broad phenotype terms. This mapping was propagated to all their descendant terms in the HPO hierarchy. Columns from left to right: (1) HPA tissue, (2) TS tissue, (3) Mapped HPO id, (4) Mapped HPO term. Last two columns are repeated for as many additional HPO terms were found.

**Supplementary Table S6. CL and UBERON mapping of HPA Cell-Types.** Each HPO Cell-Type was mapped to at least a CL term with the closest match possible. In cases where anatomical entities are defined primarily by their constituent cell types, we also provided the corresponding Uberon terms (e. g. mapping HPA “endothelial cell” to the Uberon term “Endotelium”). For 4 of the 85 HPA cell-type, neither CL terms nor Uberon terms were found (“excitatory neuron”, “mixed cell types”, “mixed immune cell” and “undifferentiated cell”).
